# GoWeb: a semantic search engine for the life science web

**DOI:** 10.1186/1471-2105-10-S10-S7

**Published:** 2009-10-01

**Authors:** Heiko Dietze, Michael Schroeder

**Affiliations:** Biotechnology Center (BIOTEC), Technische Universität Dresden, 01062 Dresden, Germany

**Keywords:** Background Knowledge, Resource Description Framework, Question Answering, Ontology Concept, Biomedical Domain

## Abstract

**Background:**

Current search engines are keyword-based. Semantic technologies promise a next generation of semantic search engines, which will be able to answer questions. Current approaches either apply natural language processing to unstructured text or they assume the existence of structured statements over which they can reason.

**Results:**

Here, we introduce a third approach, GoWeb, which combines classical keyword-based Web search with text-mining and ontologies to navigate large results sets and facilitate question answering. We evaluate GoWeb on three benchmarks of questions on genes and functions, on symptoms and diseases, and on proteins and diseases. The first benchmark is based on the BioCreAtivE 1 Task 2 and links 457 gene names with 1352 functions. GoWeb finds 58% of the functional GeneOntology annotations. The second benchmark is based on 26 case reports and links symptoms with diseases. GoWeb achieves 77% success rate improving an existing approach by nearly 20%. The third benchmark is based on 28 questions in the TREC genomics challenge and links proteins to diseases. GoWeb achieves a success rate of 79%.

**Conclusion:**

GoWeb's combination of classical Web search with text-mining and ontologies is a first step towards answering questions in the biomedical domain. GoWeb is online at: http://www.gopubmed.org/goweb

## Background

With the tremendous growth of the World Wide Web, search engines became key tools to find documents. Search engines retrieve documents for a user's keywords from a large index and rank them by various criteria. While such keyword-based search is fast and powerful to retrieve single documents, it is far from the vision of answering a user's questions by "understanding" the user's query and answers in the documents as put forward already in the late 1960s [1].

Consider e.g. a biomedical researcher, who might ask questions such as the following: Which model organisms are used to study the Fgf8 protein? Which processes are osteoclasts involved in? What are common histone modifications? Which diseases are associated with wnt signaling? Which functions does Rag C have? Which disease can be linked to fever, anterior mediastinal mass, and central necrosis? What is the role of PrnP in mad cow disease?

The Web holds answers to these questions, but classical keyword-based search is not suitable to answer them, since the keywords are required to appear literally in text. However, documents do contain statements such as e.g. "wnt signalling is linked to cancer" or "we studied fgf8 expression in Zebrafish development". If there is background knowledge that cancer is a disease and that zebrafish is a model organism, then the above questions can be answered.

The use of such knowledge is at the heart of the semantic web, which promotes the use of formal statements and reasoning to deliver advanced services not available on the Web now [2]. To facilitate machine-readability and knowledge processing, a set of standards, query languages, and the semantic stack was proposed by the World Wide Web Consortium (W3C). The stack comprises at the base unique identifiers and XML as common markup language. On top of XML, it defines the Resource Description Framework (RDF) to capture subject-predicate-object triples. Furthermore, there is the modelling language RDF Schema (RDFS) and the query language SPARQL. The basic class definitions and triples of RDF are extended at the next level by the Web ontology language (OWL), which provides description logic as modelling language and by a rule layer [3].

Besides the expressiveness of OWL, mark up for vocabularies and meta-data emerged such as Simple Knowledge Organisation Systems (SKOS) [4], Dublin Core [5], Friend of a Friend (FOAF) [6] and the Semantically-Interlinked Online Communities Project (SIOC) [7]. Additionally, there are formats to embed semantic annotations within web documents, such as embedded RDF (eRDF), Microformats [8] or Resource Description Framework in attributes (RDFa) [9].

All of the above standards serve the need to formally represent knowledge and facilitate reasoning over this knowledge. They require explicit statements of knowledge. As a consequence, the amount of such structured data is still small in comparison to the unstructured data. Thus, to support semantic search there are essentially two approaches: Those, searching structured documents and reasoning over them and those, searching unstructured documents and extracting knowledge and reasoning over it. The knowledge extraction step of the latter uses combinations of natural language processing, information retrieval, text-mining, and ontologies for the knowledge extraction.

Table 1 summarises a number of semantic search engines, which work on structured and unstructured documents. The former comprise Swoogle [10], Semantic Web Search Engine (SWSE) [11], WikiDB [12], Sindice [13], Watson [14], Falcons [15], and CORESE [16]. They include existing RDF repositories and crawl the internet for formal statements, e.g. OWL files. A search retrieves a list of results with URIs. For SWSE and Falcon the result is enriched with a description and a filtering mechanism for result types. CORESE uses conceptual graphs for matching a query to its databases. WikiDB is slightly different from the others in that it extracts formal knowledge implicit in meta tags of Wikipedia pages and converts it into RDF offering querying with SPARQL.

**Table 1:** Comparison of semantic search engines

ontologies	(1) implicit through RDF, (2) GO, (3) MeSH								
textmining	(4) NLP, (5) label extraction, (6) Ontology terminology, (7) biomedical entities, (8) Wikipedia terminology								
type of documents	(9) RDF related, (10) web pages, (11) snippets, (12) abstracts, (13) fulltext								
clustering of results	(14) RDF types, (15) extracted categories, (16) textual labels, (17) ontology, (18) answers, (19) query aspects								
result type	(20) RDF resource, (21) extracted text, (22) answer, (23) snippet, (24) sentence, (25) fulltext, (26) cluster, (27) induced ontology, (28) abstract								
Semantic Search Engines	structured/unstructured	ontologies	textmining	number of documents	type of documents	clustering of results	result type	highlighting	scientifically evaluated
Swoogle	rdf	1		≫Mio	9		20		yes
SWSE	rdf	1		≫Mio	9	14	20		yes
Sindice	rdf	1		≫Mio	9		20		yes
Watson	rdf	1		≫Mio	9		20		yes
Falcons	rdf	1		≫Mio	9	14	20	yes	yes
CORESE	rdf	1		≫Mio	9		20		yes
WikiDB	rdf	1		≫Mio	9		20		
Hakia	txt		4	≫Bio	10	15	21	yes	
START	txt		4	≫Bio	10		22		yes
Ask.com	txt		4	≫Bio	10		23		
BrainBoost	txt		4	≫Bio	10		24	yes	
AnswerBus	txt		4	≫Bio	10		25	yes	
Cuil	txt		4,8	≫Bio	10	15	21	yes	
Clusty	txt		5	≫Bio	10	16	23,26	yes	
Carrot	txt		5	≫Bio	11	16	23,26	yes	yes
PowerSet	wiki		4,8	≫Mio	10	15	23,25	yes	
QuAliM	wiki/txt		4,8	≫Mio	11,10		22		yes
GoWeb	txt	2,3	6,7,8	≫Bio	11	17	23,27	yes	yes
askMedline	xml	3		≫Mio	12		28		yes
EAGLi	xml	2	4,6	≫Mio	12	18	22,28	yes	yes
GoPubMed	xml	2,3	6,7,8	≫Mio	12	17	23,27,28	yes	yes
ClusterMed	xml	3	5	≫Mio	12	16	26,28	yes	yes
IHop	xml	3	6,7	≫Mio	12	19	24,28	yes	yes
EBIMed	xml	2,3	6,7	≫Mio	12	17	24,27	yes	yes
XplorMed	xml	3	5,6	≫Mio	12	17	21,28	yes	yes
Textpresso	xml	2	6	≫Mio	13	17	28	yes	yes
Chilibot	xml		7	≫Mio	12		24	yes	yes

As mentioned, the above systems are limited by the availability of structured documents, a problem addressed by approaches such as the semantic media wiki [17] and large efforts such as Freebase [18], which provides an environment to author formal statements. The second class of tools works on unstructured text and therefore does not suffer from this limit. The systems can be distinguished by the document source they work on (Web, Biomedical, Wiki), the use of background knowledge in the form of ontologies, the use of text-mining techniques such as stemming, concept identification, deep/shallow parsing.

Hakia, START [19], Ask.com, BrainBoost (Answers.com), AnswerBus [20], Cuil [21], Clusty [22], and Carrot [23] are engines that work on Web documents. Hakia, START and AnswerBus use natural language processing to understand documents, while Cuil, Clusty and Carrot cluster search results and aim to label clusters with phrases, which are offered as related queries. Cuil, Clusty and Carrot are not semantic search engines in a strict sense, since these phrases are not part of an ontology or vocabulary. However, they do have the benefit of being generally applicable and Cuil offers definitions for phrases where available. Ask.com uses its ExpertRank, an algorithm for computing query-specific communities and ranking in real-time, to identify relevant pages [24]. They include structured knowledge to generate answers.

BrainBoost is a meta-search engine. It uses the proprietary AnswerRank algorithm applying machine learning and natural language processing. It ranks answers extracted from the top websites.

Wikipedia is a valuable resource to answer questions and hence some engines are specifically applied to it.

PowerSet applies e.g. natural language processing to Wiki in a similar manner to Hakia. QuAliM [25] uses a pattern based approach for sentence analysis. Semantic type checking for answers and a fallback mechanism with web search is implemented in QuAliM.

The above tools are intended to be general and as a result they generally do not cover the biomedical domain well. Searching for example for a protein such as Fgf8, PowerSet and Hakia offer information on the protein, but are not able to find zebrafish as a model organism.

Engines such as askMedline, EAGLi [26], GoPubMed [27], ClusterMed, IHOP [28], EBIMed [29], XplorMed [30], Textpresso [31] and Chilibot [32] address this by processing biomedical literature in full text (Textpresso) or abstracts as available in the PubMed literature database. With a focused domain, these engines can use background knowledge. GoPubMed and EBIMed use e.g. the GeneOntology and the Medical Subject Headings, MeSH; XplorMed filters by eight MeSH categories and extracts topic keywords co-occurrences; Chilibot extracts relations and generates hypotheses; IHOP uses genes and proteins as hyperlinks between sentences and abstracts; EAGli and askMedline process questions as input for the search.

Finally, besides all of the automated approaches, Google, Yahoo! and Microsoft use humans to answer questions in their services Google Answers, Yahoo! Answers and MSN Live Search QnA.

Closely related to semantic search, is semantic hyperlinking as implemented in the Conceptual Open Hypermedia Service (COHSE). COHSE annotates a given web page with concepts and offers services based on the identified concepts [33, 34].

None of the above systems combines the simplicity of keyword search on the vast amounts of Web documents with the use of biomedical background knowledge to filter large keyword results with biomedical ontologies. Here, we address this by introducing the GoWeb search engine. GoWeb issues queries to Yahoo and indexes the snippets semantically with ontology terms. These are then offered to filter results by concepts. In order to demonstrate the power of this approach in question answering, we evaluate GoWeb on three benchmarks with questions on gene/function, symptom/disease, and protein/disease relationships and compare it to existing solutions.

## Methods

GoWeb is an internet search engine based on ontological background knowledge. It helps to filter potentially long lists of search results according to the categories provided by the GeneOntology (GO) [35] and the Medical Subject Headings (MeSH). With GoWeb one can use the GoPubMed [27] features together with the wide range of information sources available in the internet. It offers an efficient search and result set filtering mechanism, highlighting and semi-automatic question answering with the ontological background knowledge.

**Figure 1:**
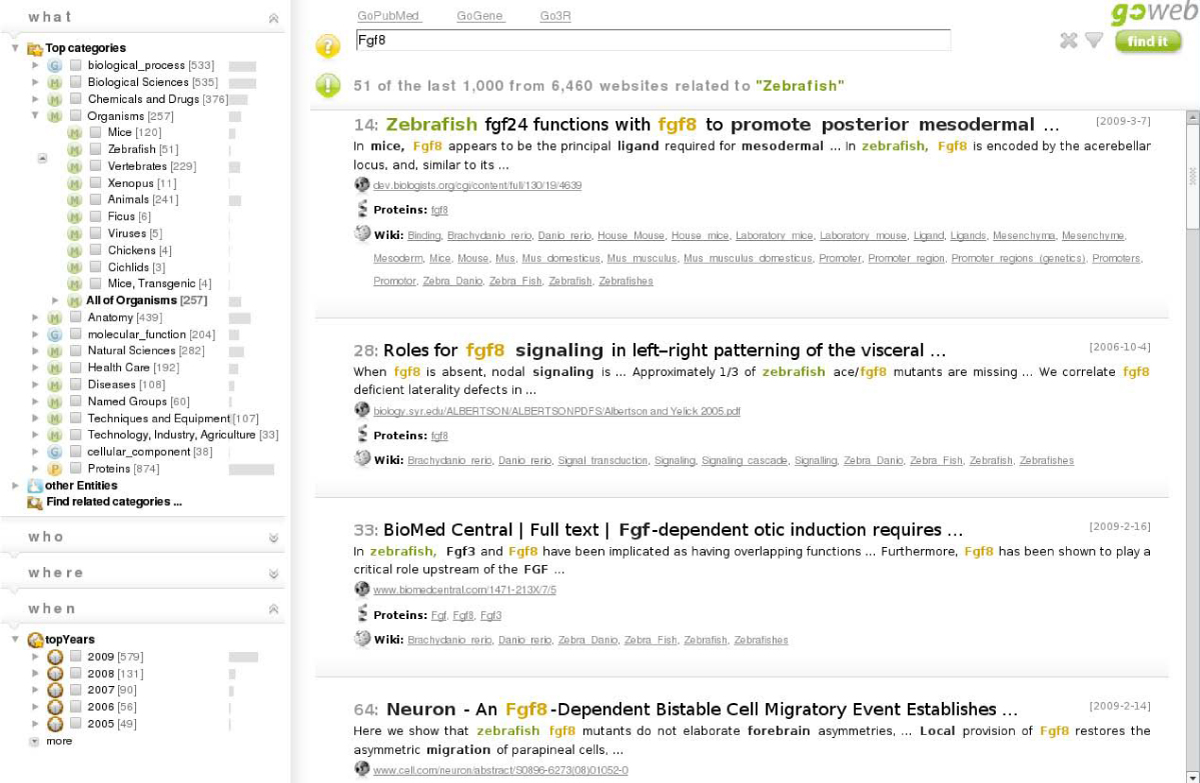
**GoWeb screen shot**. GoWeb screen shot, shown with example query Fgf8 and selected term "Zebrafish". On the left of the GoWeb website are the semantic filters in the *where*-*what*-*who*-*when* panels. In the *what* panel the GO and MeSH are show in a tree representation. For this example the MeSH branch "Organisms" is open. The most relevant concepts in this branch are listed, for instance "Mice" and "Zebrafish". The number of matching search results is given in brackets and is illustrated with a small bar chart. The bar indicates the fraction compared to the overall result set count. The wider the bar the more often it occurs in the search result. On the right side are the search results with the query field and summary on top. The search summary contains information about the current and overall number of search result. The individual search results are presented as a list. Each result has a title and a short text extract. In both keywords and terms are highlighted. The number in front of the title represents the original result position.

To facilitate the easy usage of GoWeb, the website is structured in three panels. Please consider Figure 1, a screen shot of the GoWeb web page. The left panel contains the background knowledge and other retrieved meta-data. For quicker navigation the panel is organized in the four categories *what, who, where, when* (4 w panel). The top-right panel holds the query field (search panel). In the third panel below the input field, the actual search results are presented (document panel).

The semantic filters of GoWeb are presented in the 4 w panel. The *what* category contains the result tree for the ontological background knowledge. It uses a tree representation for relations between the ontology concepts of background knowledge. In this way a concept in the tree represents the concept itself and its children. Thus, allowing the user to select relevant concepts with one click.

The *who* panel contains filters related to persons, companies or institutions. In the *where* panel there are filters related to locations, such as cities, countries and similar. The *when* category assists with time related filters, e.g. a date.

### Algorithm

The search is executed by a traditional keyword based search service. We use Yahoo! Search BOSS service [36]. The result of a submitted search is a list of textual summaries for web documents, called snippets. Next, GoWeb uses entity recognition techniques to map concepts from the background knowledge to the snippets.

The algorithm for the identification of ontological concepts in text is based on the GoPubMed algorithms [27]. For the identification of protein and gene names we use the approach by Hakenberg et al. [37], which achieved the best results in the gene identification task of BioCreAtIvE 2 (Critical Assessment of Information Extraction systems in Biology) in the year 2007. Further entity recognition services can be integrated into GoWeb. Currently the OpenCalais service [38] is used to identify names and places.

The identified entities of each result and found keywords are the basis for a co-occurrence based semantic filtering mechanism of GoWeb. The filter uses the part-of and is-a relationships from GO and the tree structure of MeSH. These relations are used to induce the relevant search result for each concept from the background knowledge. The induction result for all search results for a query is also used to select important concepts. These top concepts are selected for the entire background knowledge and for each sub category. The selection of top concepts includes the occurrence frequency, the hierarchy level and, if available, a global frequency from a pre-analyzed corpus.

### Architecture

The workflow for GoWeb can be described as follows. The user submits a query through the search form on the GoWeb website to the server. The server preprocesses the query and sends a search request to the search service. The search service returns the first results. The first results are then annotated, highlighted (concepts and keywords), rendered and sent to the user.

**Figure 2:**
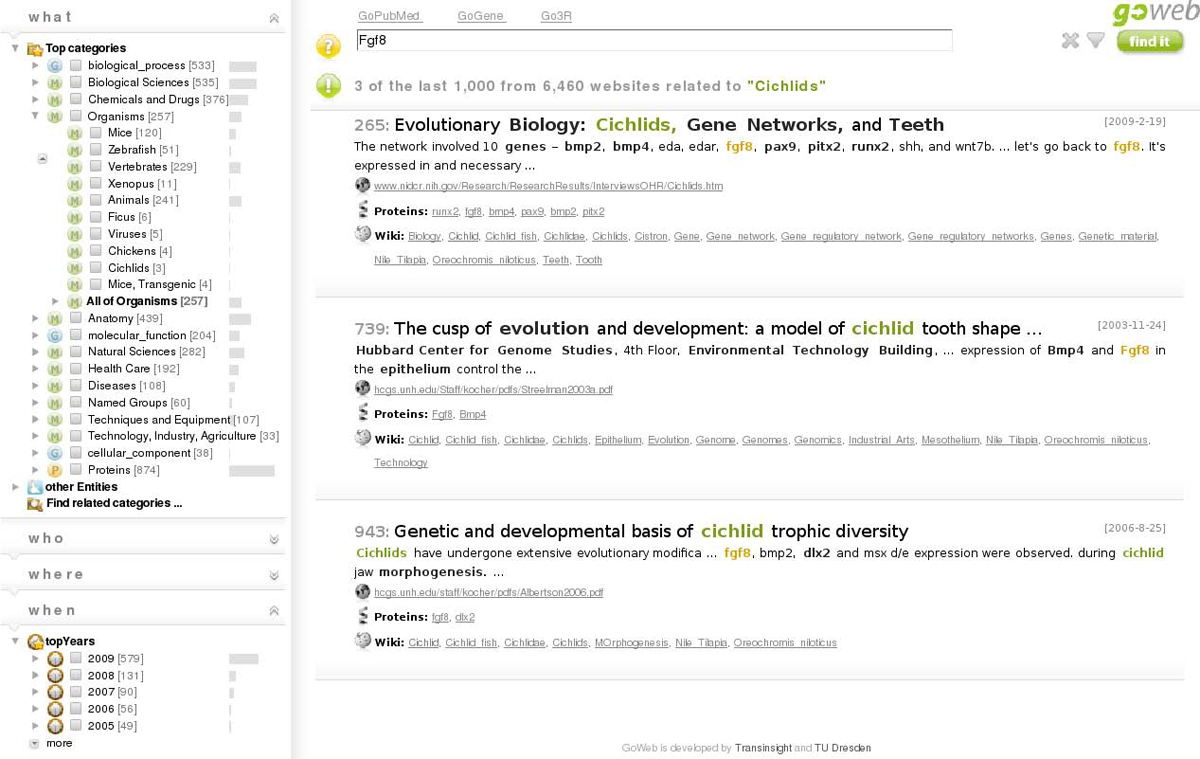
**GoWeb workflow**. General workflow for GoWeb showing the main components and the interactions between the external services. The workflow starts with the user submitting a query via the search input field from the GoWeb page (1). The search request is parsed and transformed in a search for the external Yahoo! BOSS (2). The service return a list of results, snippets. The textual content is annotated by GoWeb (3) and the additional external OpenCalais service (4). The search keywords and the identified entities form the annotation are highlighted in the search results. Then the results are rendered and sent to the browser (5). Based on the annotations and the ontology structure the tree representation is induced; top concepts are selected and sent to the browser (6).

The user can now already browse the first results. Once the first results are processed, the server starts fetching the remaining results. This is done for up to 1000 results. Then all fetched results are annotated. To reduce the response time, the fetching and annotation of results is done in parallel. The annotation information is then used to induce a tree representation and top concepts of the ontological background knowledge for the submitted query (result tree). Then this information is rendered and sent to the user-interface using AJAX (Asynchronous JavaScript and XML) technologies using a JSON (JavaScript Object Notation) based message format to reduce the required time and bandwidth. An overview is available in Figure 2.

**Figure 3:**
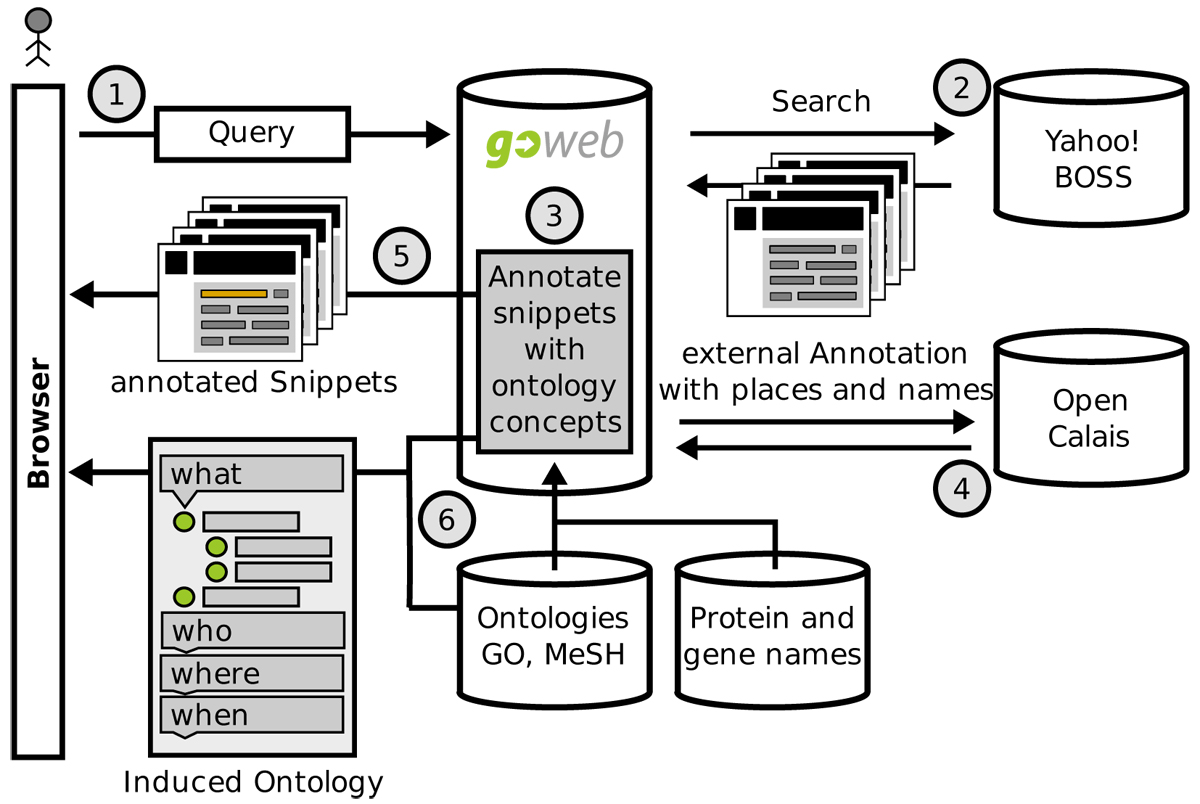
**GoWeb workflow (2)**. Workflow for a request containing a concept selected from the result tree in the user browser. When a user clicks on a concept in tree from the GoWeb website (1), the browser sends a request to update the search results (2). GoWeb filters all search results. If a result is annotated with the concept or a child of this concept, it is included in the new result list (3). Additionally the results are reranked and the highlighting of the selected concept and children is updated. The new result list is finally send to the browser.

If the user selects a concept in the result tree by clicking it, a request is made to update the presented documents. This includes a filtering step of the result set and a re-ranking step. For an illustration of this workfow see also Figure 3. The new ranking is based on the found concepts, keywords and the original ranking. A distance measure between keywords and selected concept is included for the reranking calculation.

Once the user has decided to open a web page, GoWeb offers to highlight the page with the keywords and concepts from the background knowledge. This is done with a proxy-based solution. The server checks if this page is annotatable, e.g. the content is HTML based. Then the GoWeb-server fetches the site and analyzes the content, adds the annotations and sends the result to the user. If the content is not processable by the proxy there is an automatic forward to the original content.

## Results and discussion

The goal of GoWeb is to use ontologies and text-mining in semantic web search to answer questions. Here, we give some examples and evaluate the question answering capabilities of GoWeb on three benchmarks.

In the introduction we raised questions such as the following: *Which model organisms are used to study the Fgf8 protein? Which processes are osteoclasts involved in? What are common histone modifications? Which diseases are associated with wnt signaling? In which countires the chagas disease is most prominent?*

An answer to these questions can be found using GoWeb. For example Fgf8 is studied in Mice, Zebrafish; osteoclasts are involved in bone resorption; common histone modifications are Methylation and Acetylation; the wnt signaling pathway is associated with neoplasms like breast cancer, tumors or leukemia and chagas is most prominent in Brazil, Argentina, Bolivia and other Latin American countries.

**Figure 4:**
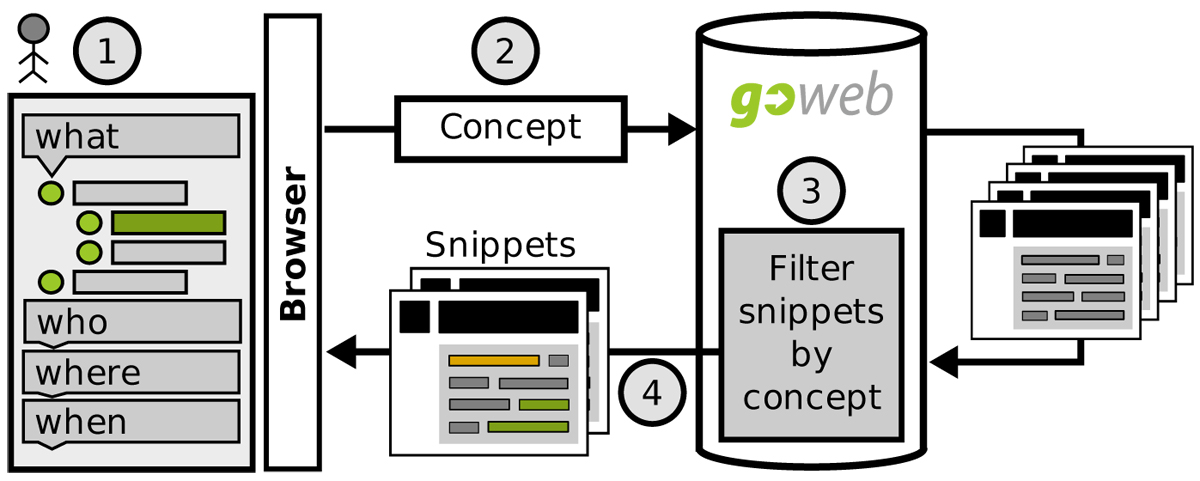
**GoWeb screen shot**. GoWeb screen shot, shown with example query Fgf8 and selected term "Cichlids". Next to the top terms as answers there is for instance the concept "Cichlids" listed under "Organisms". When selected GoWeb retrieves the matching snippets. For this example search the result set is reduced to three articles, which were formerly on positions 265, 739 and 943. The snippets talk about the usage of cichlids in the study of tooth and jaw morphogenesis. To learn more about cichlids, there are the concept definitions available in the tooltips or the exploration of related links to Wikipedia.

The answers were directly obtained with GoWeb using simple keyword searches and the induced background knowledge. For example the answer to the first question can be found in the following way: first submit the query Fgf8 the answer is directly shown as listed concepts in the organism's part of the background knowledge (see also Figure 1 and 4). To retrieve the corresponding search results click on the organism. To answer the last question the approach is very similar. Submit the query keyword chagas. Open the *where* panel and the entities and countries. There is a list of identified countries which are mostly located in Latin America. Once again a click on the country of interest in the tree retrieves the relevant articles.

The simple strategy of using keywords and filter with the induced background knowledge can be generalised to support semi-automatic question answering. Next, we will demonstrate this using three independent benchmarks.

### Genes and functions

The first benchmark is based on the association of Genes and their functions. The BioCreAtIvE 1 (Task 2) [39] was a competition for text-mining algorithms to find functional annotations in the form of GeneOntology (GO) concepts for genes in a given full-text corpus. This task is a key problem for annotators of many databases and a key question for biologists encountering a novel gene/protein, they are not familiar with.

The test set for GoWeb now contains all GO annotations and genes from the competition, which were labeled as identified with high confidence in the results. This yields a list of 457 gene names with a total of 1352 GO concepts. For example for "Rag C" there are 10 annotations: cytoplasm, small GTPase mediated signal transduction, RNA splicing, transcription, GDP binding, protein heterodimerization activity, small monomeric GTPase activity, heterotrimeric G-protein complex, protein binding and nucleus.

For a test run GoWeb was given a gene name as query. Then it is checked if the induced ontology tree contained the concepts corresponding to the expected functional annotations for the gene. For all 457 submitted names the search returned documents and the GoWeb system could identify GO concepts from these snippets. The results show that for 58.1% (785 of 1352) of the benchmark concepts are contained in the tree (recall). As the original BioCreAtIvE task specified a corpus and not an internet wide search as with GoWeb, precision is not applicable for this evaluation case.

### Symptoms and diseases

The second benchmark demonstrates the capabilities of GoWeb concerning the association of symptoms and diseases as carried out by general practitioners and medical researchers. It is based on the study by Tang et al. [40], who used a set of 26 diagnostic cases published in the case records of the New England Journal of Medicine. The symptoms were used as keywords for the search. From the search results, they proposed a possible diagnosis. For example for the symptoms "fever, anterior mediastinal mass and central necrosis", they expected to find the diagnosis "Lymphoma". With their Google-based approach the proposed diagnosis was for 15 out of 26 (58%) cases correct. It also has to be remarked, that Tang et al. is a controversial [41–43] article. One of the main issues was a possible wrong impression to the patients. It has to be clear that a search can not replace the professional and trained diagnostic capabilities of a physician. Especially in the medical domain web search results have to be handled with careful considerations.

In the experimental setup for GoWeb the same keywords as in the original paper, were used. Each diagnosis has been mapped to the corresponding MeSH concept, if possible. Then in the experiment a query was given to the GoWeb system and the resulting induced background knowledge tree was evaluated. As an additional comparison for GoWeb we also applied this benchmark and experimental setup to the GoPubMed system [27].

GoWeb can provide the correct answer in 20 out of 26 (77%) cases. In 10 of these cases, the answer term is found directly in the top categories of the Diseases subtree of MeSH (see Table 2). With up to 10 categories per subtree, this equals to a top 10 ranking or better in these cases for the identified ontology concepts. The cases 8, 10 and 18 are not marked as successful, although the results mention the searched concepts. But they all find only one article, the article [40] this analysis relies on. With GoPubMed an answer could only be found in 13 cases. GoPubMed searches only in scientific abstracts and does not include web contents such as clinical trails, general health pages, disease group pages, etc. For a comparative overview see Table 3.

**Table 2:** Overview of the GoWeb results for the symptoms and diseases benchmark

	Query	GoWeb	Count
5	Acute "Aortic regurgitation" depression abscess	Tree: Endocarditis, Bacterial	7 (1000)
6	oesophageal cancer hiccup nausea vomiting	Tree: Adenocarcinoma AND Intestinal Obstruction	2 (1000)
7	hypertension "adrenal mass"	Top categories: Cushing Syndrome	41 (1000)
8	"hip lesion" child	no, bmj article	258
9	HRCT centrilobular nodule "acute respiratory failure"	Finds the case studies this analysis relies on	15
10	fever bilateral "thigh pain" weakness	no, bmj article	500
11	fever "anterior mediastinal mass" central necrosis	Top categories: Lymphoma	66 (323)
12	multiple "spinal tumors" "skin tumors"	Top categories: Neurofibromatoses	21 (240)
14	"ulcerative colitis" "blurred vision" fever	Tree: Vascultits	2 (1000)
15	nephrotic syndrome "Bence Jones" ventricular failure	Top categories: Amyloidosis	20 (247)
16	hypertension papilledema headache "renal mass"	Tree: Pheochromocytoma	1 (31)
17	"sickle cell" pulmonary infiltrates "back pain"	Top5 snippet is ACS	1000
18	fibroma astrocytoma tumor leiomyoma scoliosis	no, bmj article	1 (47)
19	pulmonary infiltrates "cns lesion" OR "Central nervous system lesion"	no	87
22	CLL encephalitis	Tree: West Nile Fever	3 (1000)
25	"portal vein thrombosis" cancer	Tree: Phlebitis	9 (1000)
26	"cardiac arrest" exercise young	top categories: Cardiomyopathy, Hypertrophic	22 (1000)
27	ataxia confusion insomnia death	Tree: CJD	17 (1000)
28	ANCA haematuria haemoptysis	Top categories: Churg-Strauss Syndrome	3 (126)
29	myopathy neoplasia dysphagia rash periorbital swelling	Top categories: Dermatomyositis	4 (32)
30	"renal transplant" fever cat lymphadenopathy	Top categories: Cat-Scratch Disease	13 (322)
31	"buttock rash" "renal failure" edema	no	120
33	polyps telangiectasia epistaxis anemia	Top categories: Telangiectasia, Hereditary Hemorrhagic	33 (1000)
34	"bullous skin" "respiratory failure" carbamazepine	Top categories: Epidermal Necrolysis, Toxic	4 (25)
36	seizure confusion dysphasia lesions	no	1000
37	cardiac arrest sleep	Tree: Brugada Syndrome	3 (1000)

**Table 3:** Comparison of Google, GoPubMed and GoWeb for symptoms and diseases benchmark

Case	5	6	7	8	9	10	11	12	14	15	16	17	18	19	22	25
Google	✓		✓	✓	✓		✓	✓		✓		✓				
GoPubMed	✓	✓	✓					✓	✓			✓				✓
GoWeb	✓	✓	✓		✓		✓	✓	✓	✓	✓	✓			✓	✓
Case	26	27	28	29	30	31	33	34	36	37	Count			Ratio		
Google	✓	✓	✓	✓	✓		✓	✓		✓	16			62%		
GoPubMed	✓		✓		✓		✓	✓		✓	13			50%		
GoWeb	✓	✓	✓	✓	✓		✓	✓		✓	20			77%		

For example for the case study number 28 GoWeb finds 126 articles for the query "ANCA haematuria haemoptysis". Under diseases one can find the MeSH concept "Churg-Strauss Syndrome". A click on the concepts in the tree retrieves three snippets containing the concept. The resulting snippets are:

- **Laboratory imposed restrictions on ANCA testing – 63 (5): 594 – Annals of the Rheumatic Diseases**

The laboratory has performed ANCA testing only when the request form indicated ... haematuria (requests from the renal/transplant unit), Churg-Strauss syndrome, ...

http://ard.bmj.com/cgi/content/extract/63/5/594

- include haemoptysis (13% of patients), cystic bone lesions (4–20% of ... Some 70–75% of patients with Churg-Strauss syndrome have ANCA ...

http://www.hospitaldoctor.ie/hospital_doctor/pdfs/HOS_DOC_MARCH_APRIL_05.pdf

- **Churg-Strauss Syndrome – Patient UK**

Pulmonary: asthma, pneumonitis and haemoptysis ... patients are perinuclear-ANCA (p-ANCA) positive (antimyeloperoxidase antibodies) ...

http://www.patient.co.uk/showdoc/40024815/

The GoWeb system performs better than GoPubMed because the underlying search engine has a larger repository of documents. Additionally, it can also index the full text, if it is available on the web. The MEDLINE search for all PubMed based search engines, like GoPubMed, is only based on abstracts. This corresponds with the fact that the MEDLINE search returns often none or only one article abstract.

### Proteins, diseases and evidences

Linking proteins and disease is a key task for many molecular biomedical researchers. The third benchmark for GoWeb is based on the questions from the TREC Genomics Track 2006 [44]. The results of TREC Genomics Track 2006 comprise a benchmark that focused on passage retrieval for question answering. It is based on full-text documents from the biomedical literature. For the year 2006 there were 28 questions. With GoWeb one can answer 22 of these 28 questions (78,6%). In 13 of these cases the semantic filter helped to reduce the result set. For a summary of all questions please have a look at Table 4.

**Table 4:** Summary of TREC Genomics 2006 answering capabilities of GoWeb

Question	160	161	162	163	164	165	166	167	168	169
Answered	✓	✓	✓	✓	✓	✓	✓	✓	✓	✓
Filter	✓	✓	✓		✓	✓				
Question	170	171	172	173	174	175	176	177	178	179
Answered			✓	✓	✓	✓	✓	✓		
Filter					✓		✓	✓		
Question	180	181	182	183	184	185	186	187	Count	
Answered	✓	✓	✓	✓		✓	✓		22	
Filter	✓		✓	✓		✓	✓		13	

For GoWeb the questions were transformed into keywords. The complete listing of questions and keywords is available in Table 5. A question was marked as successfully handled, if there was a snippet that contained a valid answer. The answers had to be available in the top 20 search results. The second aspect addressed with this benchmark was to show the capabilities of the filtering feature. Filtering by background knowledge helps to reduce large results to a smaller set of relevant documents. It was marked as applied, if the answers were found by using the filtering feature. If the semantic filter feature was used the new top 20 after filtering and re-ranking were checked. In most of the cases the re-ranking shifted a valid answer and evidence in the top 5 or better. Similar to the first benchmark, precision is not applicable. The gold-standard for the TREC Genomics Track 2006 contains only passages from the original corpus and competition. New answers and answers from other sources are not comparable.

**Table 5:** TREC Genomics 2006 questions and keywords

160	What is the role of PrnP in mad cow disease?	PrnP
161	What is the role of IDE in Alzheimer's disease?	IDE Alzheimer
162	What is the role of MMS2 in cancer?	MMS2
163	What is the role of APC (adenomatous polyposis coli) in colon cancer?	APC adenomatous polyposis coli
164	What is the role of Nurr-77 in Parkinson's disease?	Nurr-77
165	How do Cathepsin D (CTSD) and apolipoprotein E (ApoE) interactions contribute to Alzheimer's disease?	"Cathepsin D" "apolipoprotein E"
166	What is the role of Transforming growth factor-beta1 (TGF-beta1) in cerebral amyloid angiopathy (CAA)?	TGF-beta1 cerebral amyloid angiopathy
167	How does nucleoside diphosphate kinase (NM23) contribute to tumor progression?	NM23 tumor progression
168	How does BARD1 regulate BRCA1 activity?	BARD1 BRCA1
169	How does APC (adenomatous polyposis coli) protein affect actin assembly?	adenomatous polyposis coli actin assembly
170	How does COP2 contribute to CFTR export from the endoplasmic reticulum?	COP2 CFTR
171	How does Nurr-77 delete T cells before they migrate to the spleen or lymph nodes and how does this impact autoimmunity?	Nurr-77 T cell
172	How does p53 affect apoptosis?	p53 apoptosis
173	How do alpha7 nicotinic receptor subunits affect ethanol metabolism?	alpha7 nicotinic receptor ethanol
174	How does BRCA1 ubiquitinating activity contribute to cancer?	BRCA1 ubiquitinating
175	How does L2 interact with L1 to form HPV11 viral capsids?	L1 L2 HPV11
176	How does Sec61-mediated CFTR degradation contribute to cystic fibrosis?	Sec61 CFTR
177	How do Bop-Pes interactions affect cell growth?	Bop Pes cell growth
178	How do interactions between insulin-like GFs and the insulin receptor affect skin biology?	insulin-like GF insulin receptor
179	How do interactions between HNF4 and COUP-TF1 suppress liver function?	HNF4 COUP-TF1
180	How do Ret-GDNF interactions affect liver development?	Ret GDNF liver
181	How do mutations in the Huntingtin gene affect Huntington's disease?	Huntingtin gene
182	How do mutations in Sonic Hedgehog genes affect developmental disorders?	Sonic Hedgehog gene
183	How do mutations in the NM23 gene affect tracheal development?	NM23 tracheal development
184	How do mutations in the Pes gene affect cell growth?	Pes gene cell growth
185	How do mutations in the hypocretin receptor 2 gene affect narcolepsy?	hypocretin receptor 2 narcolepsy
186	How do mutations in the Presenilin-1 gene affect Alzheimer's disease?	Presenilin-1 Alzheimer
187	How do mutations in familial hemiplegic migraine type 1 (FHM1) gene affect calcium ion in ux in hippocampal neurons?	FHM1 calcium neuron

The answers for the first four questions (160–164) are shown in Table 6. They also demonstrate what kind of textual evidence GoWeb can provide as answers. The answer to question 160 (*What is the role of PrnP in mad cow disease*), for instance, was found by submitting the keywords and selecting the MeSH concept "Encephalopathy, Bovine Spongiform" (mad cow disease is a synonymous label for the concept) as semantic filter. The selected answer was now in the first part of the remaining relevant results. The given number 378 corresponds to the original position. This demonstrates that without the filter this answer would not have been found normally [45]. For the question 161 the keywords were specific enough. This corresponds with the original rank of first and second position for the answers.

**Table 6:** Answers for TREC Genomics 2006 questions 160 to 164

Concept	original Pos	Evidence
160 Encephalopathy, Bovine Spongiform	**378:**	Transmissible Spongiform Encephalopathy (BSE) Bovine spongiform encepalopathy is a transmissible, ... Mutations in the PRNP gene cause prion disease. ... http://www.answers.com/topic/spongiform-encephalopathy
161	**1:**	Insulin-Degrading Enzyme as a Downstream Target of Insulin Receptor ... effect relationship between insulin signaling and IDE upregulation. ... P85) was correlated with reduced IDE in Alzheimer's disease (AD) brains and in ... http://alzheimer.neurology.ucla.edu/pubs/IDEzhao.pdf
	**2:**	Insulin degrading enzyme – Wikipedia, the free encyclopedia 1 IDE and Alzheimer's Disease. 2 IDE Structure and Function. 3 References. 4 External links ... between IDE, A*β* degradation, and Alzheimer's disease. ... http://en.wikipedia.org/wiki/Insulin_degrading_enzyme
162 DNA Damage	**41:**	... concerted action of RAD5 with UBC13 and MMS2 in DNA damage repair is given by ... Finally, it is shown that MMS2, like UBC13 and many other repair genes, is ... http://db.yeastgenome.org/cgi-bin/reference/reference.pl?dbid=S000061270
163	**1:**	The official name of this gene is "adenomatous polyposis coli." APC is the gene's official symbol. ... adenomatous polyposis – caused by mutations in the APC ... http://ghr.nlm.nih.gov/gene=apc
164 Parkinson Disease	**40:**	The aetiology of idiopathic Parkinson's disease Nurr 1 was first recognised as a transcription factor that was primarily ... Its close structural relation to Nur 77 led to its identification in stimulated ... http://www.pubmedcentral.nih.gov/articlerender.fcgi?artid=1187126
	**132:**	Concise Review: Therapeutic Strategies for Parkinson Disease Based on ... nuclear related receptor 1 (Nurr-1), thereby withdrawing the cells of the cell ... in the SVZ and the substantia nigra of the healthy adult rat brain [77, 98] ... http://stemcells.alphamedpress.org/cgi/content/full/25/2/263
	**221:**	Parkinson's disease: piecing together a genetic jigsaw – Dekker et al ... study decreased rapidly with later onset: 77% of patients with onset of disease ... agenesis of mesencephalic dopaminergic neurons in Nurr-1 deficient mice. ... http://brain.oxfordjournals.org/cgi/content/full/126/8/1722

There are two main reasons for GoWeb to not be able to find an answer for all question in the benchmark. The first is that the question is too complex. The answer is too long to be formulated in a sentence or snippet. For example the question 171 contains actually two questions. The second reason is that the question domain is not sufficiently modelled in the background knowledge. For question 178, for instance, *skin biology* has no corresponding concept and is too general to be mentioned directly in text.

### Discussion and comparison

The three used benchmarks provide a basis for the evaluation of GoWeb. They demonstrate the power of the idea but also its limitations. The starting point is the usage of snippets. This is already a limitation in terms of completeness. Snippets can be seen as an abstraction. They try to summarize facts related to the keywords. A snippet might be too short to contain very complex facts, some information will be lost. But important information is more likely to be in the snippet, because important facts are often close to each other in the original text. Thus it is more likely to be also contained in the snippet. The co-occurrence is used as approximation for relation extraction. One advantage of using this simpler approach is the reduced computational complexity. A proper natural language processing (NLP) based approach would need more computational time. Furthermore, NLP might be hindered by the unpredictable grammatical structure of snippets. With GoWeb the complete annotation for 1000 results can be done on-the-fly and still provides response time of a few seconds.

The decision to use the Yahoo! BOSS API as search service was made on a technical level. The Yahoo! API offers to retrieve the most results per requests, parallel requests, unlimited queries per day and allows re-sorting of results. The latter is explicitly not allowed for the Google AJAX Search API in their current terms of service. The same goes also for the Microsoft Live Search API. Other APIs such as Amazon's Alexa Web Search or the Google SOAP Search API are deprecated and will be discontinued.

If more information than the snippet is required, it is necessary to fetch the web pages and analyze them. This could be done on runtime for the result set or pre-calculated during a crawl of the internet. Both options have major drawbacks. Fetching and analyzing of web pages on-the-fly is not feasible with the requirement of a short response time. The crawling of the internet is possible, but requires a significant amount of resources in terms of hardware and bandwidth to keep the index up-to-date. This is demonstrated by the popular search engines. Each of them uses several data and computing centers. Although the search requests from the user are the main load, keeping the index up-to-date is an important aspect. One advantage of a separate crawl is the chance to build a semantically enhanced index. Such an enhanced index offers the option to include concepts directly into the search and not as post-processing step like GoWeb.

To include all information from a web page will increase the recall. But it would also increase the problem of false positives from matching errors or irrelevant parts. The false positives would also unnecessarily increase the size of an index. With the option to pre-process the information, e.g. with topic recognition or disambiguation algorithms, this can be compensated. For a specialized system with a limited number of documents and known document structure, a semantic index might be a better solution than GoWeb. The application of text mining for concept identification is important for finding the relevant snippets in the search results. A simple keyword can not easily replace the additional information from the background knowledge. This includes synonymous labels and related concepts. For example for heart diseases in MeSH there are over 570 related labels. Although a user can try to emulate this behavior by using long Boolean queries, there is a prerequisite. The user has to know them beforehand. This expert knowledge is compressed and available by using ontology concepts.

The types of questions handled best by GoWeb have to be transformable into keywords and concepts. The answer provided by GoWeb will be either an inferred concept or a sentence/short text extract in the snippet. These options reduce the types of questions which can be answered by GoWeb. For example in a question classification by Tomuro and Lytinen [46] GoWeb performs best with questions of type definition ('What does X do?'), reference ('What', 'Which') or entities ('Who'). But it can not answer question types like manner of action, degree or interval ('how many', 'how much' or 'how long', e.g. What percentage of children are vaccinated?) and procedure ('how to'). For a medical question taxonomy by Ely et al. [47], GoWeb works with questions related to diagnosis branch but it fails for questions from the treatment branch (What are the options for treatment of condition y in situation z?), management (What is the best way to discuss or approach discussion of difficult issue x?) and nonclinical (What are the legal considerations in situation y?).

In comparison to existing systems which are mainly focusing on searching OWL and RDF content (e.g. Swoogle, SWSE) GoWeb covers a broader area. Current RDF search engines cover millions of RDF statements, whereas the internet search engines cover billions of websites. Unfortunately, most of the information in websites is unstructured text. GoWeb tries to bridge the semantic gap with the limited amount of available semantic annotations by employing text-mining for extraction of ontology concepts from text. In a nutshell, GoWeb exploits that keywords and ontology terms co-occurring in snippets are often facts.

Traditional search engines like Google have the coverage but they miss the explicit usage of ontological background knowledge. They only present a long list of results. This works very well for simple retrieval of documents, but has limits for more complex task, e.g. answering questions. Here the semantic filtering with concepts as in GoWeb helps to reduce the result list to relevant answers. If a snippet does not contain the relevant terms, it is likely to be not relevant.

In comparison with other internet search based systems like Hakia or PowerSet the advantage of GoWeb is its additional background knowledge for the biomedical domain. Only GoWeb combines the usage of the GeneOntology (GO), the Medical Subject Heading (MeSH) and protein identification. A clustering of text labels like Clusty or Carrot can not replace the structural knowledge of an ontology. In comparison to PubMed-based systems GoWeb can index the additional resources of full text articles on the web.

## Conclusion

The search interface of GoWeb provides with the *what*-*where*-*who*-*when* categories a simple way to browse the results. Next to the actual search results GoWeb offers also additional information like definitions of concepts or Wikipedia links.

Together with the filtering mechanism to reduce the result set from 1000 possible results to a small number of relevant entries GoWeb offers powerful tool for semantic search in the biomedical domain. Overall, the paper shows that co-occurrences of keywords and ontology terms in lists of snippets are often approximating facts, which aid to answer questions. The simplicity of the approach ensures scalability and speed, still achieving success rates of up to 80%.

## Availability and requirements

GoWeb is online available under the URL http://www.gopubmed.org/goweb/. The GoWeb website has been tested with the following web browsers:

- Firefox 2.0, 3.0
- Internet Explorer 6.0, 7.0
- Safari 3.2

The usage of GoWeb is free of charge for non-commercial users. For commercial users some restrictions may apply. For automated querying from any user, for example using programs, a registration is strongly recommended.

## Abbreviations

BioCreAtivE: Critical Assessment of Information Extraction systems in Biology
GO: GeneOntology
MeSH: Medical Subject Headings
RDF: Resource Description Framework
SPARQL: SPARQL Protocol and RDF Query Language
TREC: Text REtrieval Conference
XML: Extensible Markup Language.
